# Histological Assessment of the Bile Duct before Liver Transplantation: Does the Bile Duct Injury Score Predict Biliary Strictures?

**DOI:** 10.3390/jcm12216793

**Published:** 2023-10-27

**Authors:** Mark Ly, Ngee-Soon Lau, Catriona McKenzie, James G. Kench, Doruk Seyfi, Avik Majumdar, Ken Liu, Geoffrey McCaughan, Michael Crawford, Carlo Pulitano

**Affiliations:** 1 9E Australian National Liver Transplantation Unit, Royal Prince Alfred Hospital, Missenden Rd., Camperdown, Sydney, NSW 2050, Australia; mark.ly@sydney.edu.au (M.L.);; 2 Faculty of Medicine and Health, The University of Sydney, Sydney, NSW 2050, Australia; 3 Department of Tissue Pathology and Diagnostic Oncology, Royal Prince Alfred Hospital, Sydney, NSW 2050, Australia; 4 Centenary Institute, Sydney, NSW 2050, Australia

**Keywords:** liver transplantation, biliary complications, bile duct injury, organ preservation, organ donation

## Abstract

Introduction: Histological injury to the biliary tree during organ preservation leads to biliary strictures after liver transplantation. The Bile Duct Injury (BDI) score was developed to assess histological injury and identify the grafts most likely to develop biliary strictures. The BDI score evaluates the bile duct mural stroma, peribiliary vascular plexus (PVP) and deep peribiliary glands (DPGs), which were correlated with post-transplant biliary strictures. However, the BDI score has not been externally validated. The aim of this study was to verify whether the BDI score could predict biliary strictures at our transplant centre. Methods: Brain-dead donor liver grafts transplanted at a single institution from March 2015 to June 2016 were included in this analysis. Bile duct biopsies were collected immediately before transplantation and assessed for bile duct injury by two blinded pathologists. The primary outcome was the development of clinically significant biliary strictures within 24 months post-transplant. Results: Fifty-seven grafts were included in the study which included 16 biliary strictures (28%). Using the BDI score, mural stromal, PVP and DPG injury did not correlate with biliary strictures including Non-Anastomotic Strictures. Severe inflammation (>50 leucocytes per HPF) was the only histological feature inversely correlated with the primary outcome (absent in the biliary stricture group vs. 41% in the no-stricture group, *p* = 0.001). Conclusions: The current study highlights limitations of the histological assessment of bile duct injury. Although all grafts had bile duct injury, only inflammation was associated with biliary strictures. The BDI score was unable to predict post-transplant biliary strictures in our patient population.

## 1. Introduction

Biliary strictures are a common cause of morbidity after liver transplantation and can lead to re-transplantation [1]. Histological bile duct injury during organ retrieval and preservation is a major contributing factor to post-transplant biliary strictures [1,2,3]. The biliary epithelium is particularly vulnerable to ischaemic and bile-salt-mediated insults during organ preservation [2,4,5]. Although minor injury may heal through re-epithelialisation, more severe bile duct injury results in healing via fibrosis and stricture formation [3]. However, biliary strictures develop weeks to years after transplantation, which increases the need to assess bile duct injury during organ preservation [6,7]. An early assessment of bile duct injury may enable clinicians to identify grafts at high risk of biliary strictures and facilitate discard prior to transplant [2,8,9].

The Bile Duct Injury (BDI) score, described by Op Den Dries et al. in 2014 [2], uses histology to assess the biliary tree injury before transplantation [10,11]. Op Den Dries et al. systematically assessed seven features of bile duct injury (Table 1) on biopsies collected prior to graft implantation. Three features of bile duct injury, mural stromal, peribiliary vascular plexus (PVP) and deep peribiliary gland (DPG) injury, were associated with biliary strictures and formed the BDI score. The BDI score has been used to identify grafts at high risk of post-transplant biliary stricture and to compare the efficacy of interventions to preserve the biliary tree [11,12,13,14,15]. However, the score has never been externally validated. The aim of this study was to verify whether the BDI score could predict clinically significant biliary strictures after liver transplantation using Brain-dead donors (BDD) at an external institution.

## 2. Methods

### 2.1. Patients

Adult patients undergoing liver transplantation using BDD grafts at the Australian National Liver Transplantation Unit from March 2016 to June 2017 were prospectively included in the study. Donation after Circulatory Death (DCD) grafts, split livers and grafts retrieved by interstate teams were excluded from the study. Informed consent was obtained from recipients and the study was performed in accordance with the ethical guidelines of the Declaration of Helsinki. Ethics approval was obtained from the Sydney Local Health District Ethics Committee (X15-0444).

### 2.2. Graft Retrieval

Grafts were retrieved using standard donor procurement techniques as we have previously described [16,17]. Briefly, aortic-only cold perfusion was performed initially with modified Marshall solution (Soltran, Baxter Healthcare, Norfolk UK), followed by University of Wisconsin solution (UW) (Belzer UW, Bridge to Life, Northbrook, IL, USA) and transported in cold UW storage [17]. All grafts received gallbladder irrigation during donor procurement and received a bile duct flush with UW after donor hepatectomy.

### 2.3. Bile Duct Injury Scoring

**Figure 1:**
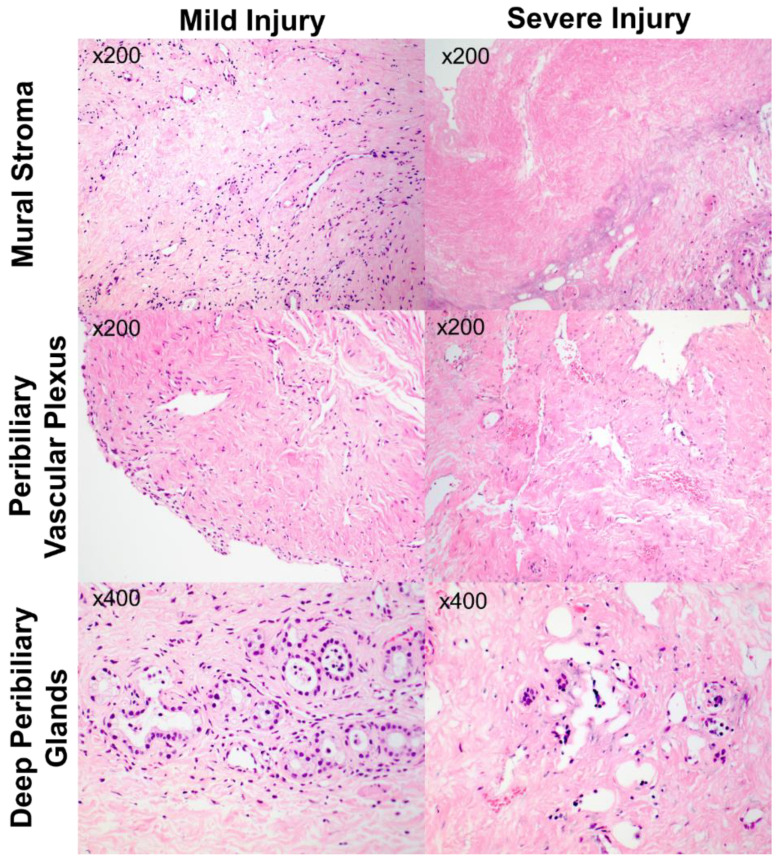
Illustrations of mild and severe injury according to components of the bile duct injury score (mural stroma, peribiliary vascular plexus and deep peribiliary gland). Magnification is provided per illustration.

Biopsies of the distal bile duct were collected at the end of cold storage during the back-table preparation of the liver. Specimens were fixed in 10% neutral buffered formalin and embedded in paraffin. Representative sections were stained with haematoxylin and eosin and assessed by two independent pathologists, blinded from clinical outcome (CM and JK). Scoring was performed according to histological definitions described by Op Den Dries et al. and Hansen et al. (Table 1). The deep peribiliary glands were assessed separately from periluminal glands and located between the stroma and muscular layer. The inflammation score was calculated using the high-powered field with the most lymphocytes and granulocytes per biopsy. Discrepancies in the assessment were resolved with multi-header consensus. Representative images of mild and severe grades of histological injury are demonstrated in Figure 1.

### 2.4. Clinical Outcomes and Definitions

Donor, recipient and intraoperative characteristics were prospectively collected. All recipients were followed up for 24 months [18]. Biliary strictures were identified using biliary imaging and included Anastomotic Strictures (ASs) and Non-Anastomotic Strictures (NASs). ASs were defined as narrowing at the biliary anastomosis requiring intervention. NASs were defined as any narrowing of the donor biliary tree, proximal to the biliary anastomosis. Biliary imaging in the post-transplant setting was only performed in the presence of clinical or biochemical features of biliary strictures [19,20]. Post-transplant care was delivered by hepatologists who were blinded from histological scores. Cold ischaemic time (CIT) was defined as the time from donor aortic cross clamp until the time the liver graft was removed from cold static storage for recipient implantation. Warm ischaemic time (WIT) was defined as the time from the end of cold static storage to graft reperfusion. Hepatic doppler was routinely performed in the post-operative period and on discretion of the treating hepatologist. Hepatic artery complications were defined as any stenosis, thrombosis, aneurysm or occlusion of the vessel on vascular imaging. Peak serum Gamma-Glutamyl Transferase within 7 days post-transplant was also recorded [21].

### 2.5. Statistics

Statistical analysis was performed using R Studio. Grafts were grouped according to the development of a biliary stricture and subtypes. Categorical variables were analysed using Fisher’s exact test. Shapiro–Wilk testing was used to determine the normality of continuous variables. Normally distributed continuous variables were analysed using student’s *t*-test. Mann–Whitney U testing was used for non-normally distributed variables. Statistical significance was defined as a *p*-value < 0.05.

## 3. Results

### 3.1. Patient Characteristics

Bile duct biopsies were collected from 60 grafts. One recipient did not survive graft implantation and another withdrew from clinical follow up. Both cases were excluded from analysis. AS, which did not require intervention, was identified in one recipient and excluded from analysis. The final analysis was performed on 57 grafts. Two recipients were re-transplanted within the study period, one for primary non-function and one for NAS.

Donor, recipient and intraoperative characteristics are described in Table 2. The median donor age was 56 (Interquartile range (IQR) 48–63) years and donor risk index was 1.581 (IQR 1.402–1.837). The median CIT was 318 (IQR 262–387) minutes, WIT was 42 (IQR 34–59) minutes and 76% had a duct-to-duct anastomosis.

### 3.2. Clinical Outcomes

Biliary strictures developed in 16 patients (28%) which included 13 ASs (23%) and 4 NASs (7%). One recipient developed both AS and NAS. The median time to identification of AS was 3.1 months and for NAS it was 6.7 months. Demographic characteristics were stratified according to those who developed a biliary stricture and those who did not (Table 2). The median donor age in the biliary stricture group was higher compared to the no-stricture group (61 vs. 54 years, *p* = 0.018). The median donor risk index was 1.746 in grafts which developed a biliary stricture and 1.581 in the no-stricture group (*p* = 0.304). Recipient age in the biliary stricture group was significantly lower than in the no-stricture group (52.3 vs. 60.5, *p* = 0.026). There was no difference between the CIT in the biliary stricture group compared to the no-stricture group (209 min vs. 325 min, *p* = 0.894). WIT was higher in the biliary stricture group compared to the no-stricture group (50 vs. 40 min, *p* = 0.041). There was no significant difference in the interval between portal and arterial reperfusion between the two groups (31 vs. 26 min, *p* = 0.439). Post-transplant hepatic artery complications were not significantly associated with biliary strictures (31% in the biliary stricture group vs. 10% in the no-stricture group, *p* = 0.099), but were significantly higher in recipients who developed NAS (75% in the NAS group vs. 11% in the no-NAS group, *p* = 0.011).

### 3.3. Bile Duct Injury Scores

Histological evidence of bile duct injury was present in all biopsies (Table 3). Severe biliary epithelial injury (greater than 50% epithelial cell loss) was present in 74% of biopsies. Mural stromal injury occurred in 70% of bile ducts, with severe injury (grade 2–3) in 26%. Peribiliary vascular plexus (PVP) injury was present in 96% of bile ducts. Grade 2 or greater (severe injury) occurred in 23%. Deep peribiliary glands (DPGs) were identified in 53 biopsies and injury was present in 74%. Severe inflammation (Grade 2, ≥50 leucocytes per High-Powered Field (HPF)) was present in 17 specimens (29%).

**Figure 2:**
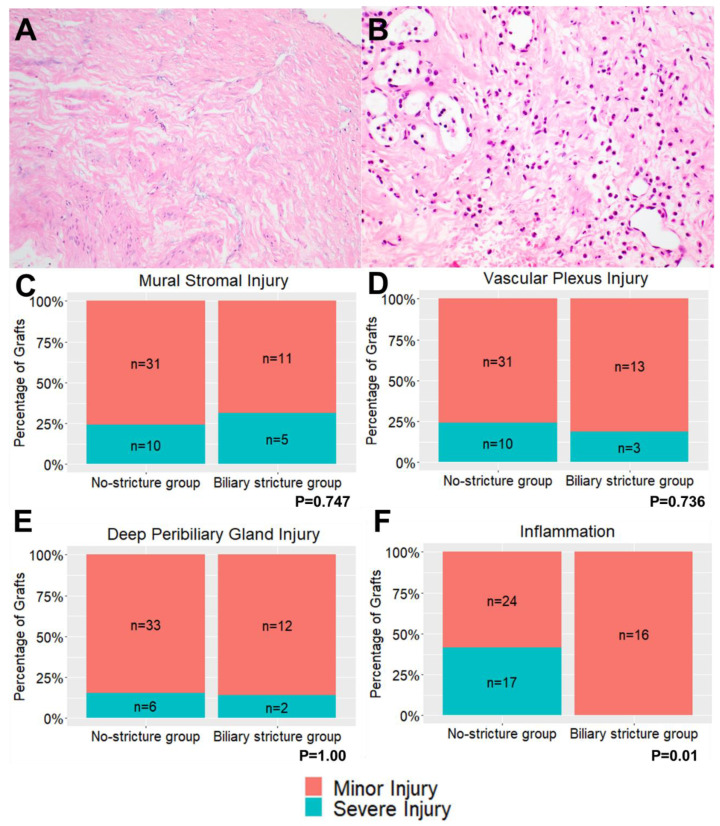
(A) Biopsy demonstrating severe mural stromal injury at ×200 magnification. (**B**) Severe inflammation at ×400 magnification. Rates of severe (**C**) mural stromal injury, (**D**) peribiliary vascular plexus injury, (**E**) deep peribiliary gland injury and (**F**) inflammation according to development of biliary strictures.

**Figure 3:**
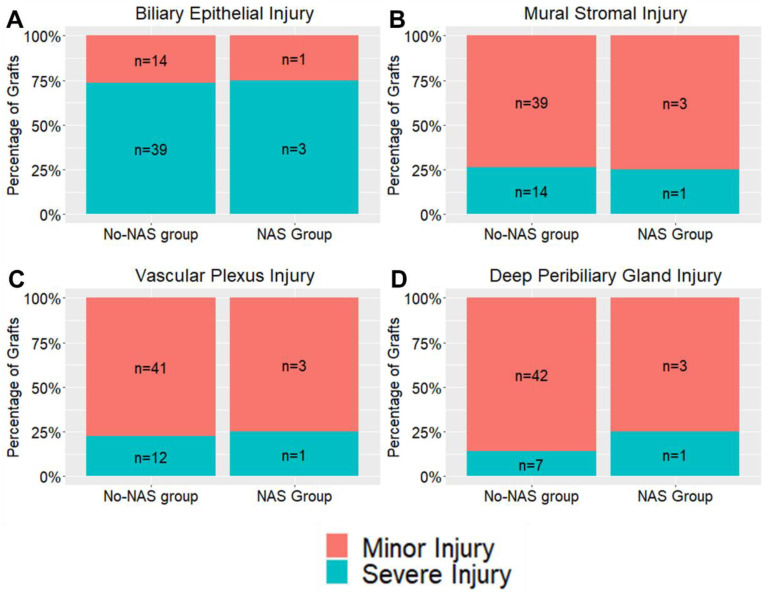
The incidence of severe injury to the (**A**) biliary epithelium, (**B**) mural stroma, (**C**) vascular plexus and (**D**) deep peribiliary glands according to development of Non-Anastomotic Strictures.

Bile duct injury scores were grouped according to the development of biliary strictures (Table 3) and were further stratified into mild or severe injury (Figure 2). Rates of biliary epithelial injury were similar between groups (*p* = 0.317). Mural stromal injury was present in 62% of the biliary stricture group and 73% of the no-stricture group. Severe mural stromal injury (grade 2–3) was present in 29% of the biliary stricture group compared to 24% of the no-stricture group (*p* = 0.7473). Severe PVP injury (grade 2–3) was similar between groups (19% in the biliary stricture group vs. 24% in the no-stricture group, *p* = 1.00). Severe DPG injury was present in 14% of grafts which developed a biliary stricture compared to 15% of grafts which did not (*p* = 0.740). Severe inflammation (≥50 leucocytes per HPF) was absent in the biliary stricture group compared to 41% in the no-stricture group (*p* = 0.001) (Figure 2F). An additional analysis was performed to compare bile duct injury in grafts which developed NAS and those which did not (Table 4). No significant differences were identified in the NAS group with respect to severe mural stromal, vascular plexus, or deep peribiliary gland injury (all *p* > 0.05) (Figure 3).

## 4. Discussion

Biliary strictures are a common cause of morbidity after liver transplantation and are thought to result from bile duct injury during organ retrieval and preservation [3,22,23]. The biliary epithelium is particularly sensitive to ischaemic insult, resulting in a variable degree of injury [2,4,24]. Severe bile duct injuries on pre-implantation biopsies have been correlated with the development of biliary strictures [2,4]. Therefore, assessments of bile duct injury could be used to identify grafts at high risk for biliary strictures, which could then receive closer surveillance or be discarded [25]. Furthermore, the development of a validated tool for assessing biliary injury could facilitate assessment of the biliary tree during ex-vivo machine perfusion [10,26,27,28].

Hansen et al. initially developed a semi-quantitative histological score to assess bile duct injury after graft reperfusion to predict biliary strictures [8]. However, Brunner et al. demonstrated that the assessment of biliary epithelial injury before graft implantation was more predictive of biliary complications than post-reperfusion biopsies [4]. Using pre-implantation biopsies of 73 patients, Op Den Dries et al. developed the BDI score to identify grafts at high risk for post-transplant NAS [2]. The BDI score evaluates three histological variables associated with NAS (mural stroma, PVP and DPG), and is commonly used to assess the biliary tree in animal and human studies [10,25,26,27,28].

The incidence and severity of bile duct injury on pre-implantation biopsies in this study were comparable to the original study by Op Den Dries et al. However, no association was observed between mural stromal, PVP or DPG injury and biliary strictures (Figure 2C–F) or NAS (Figure 3), as previously described in the BDI score. In our study, the absence of severe inflammation (≥50 leucocytes per HPF) was the only histological feature associated with the development of biliary strictures. No graft in the biliary stricture group had severe grade inflammation (Figure 2F). These findings may suggest a protective role of immune cells in the development of biliary strictures [3,29,30,31]. However, the role of inflammation in the development of post-transplant biliary strictures requires further clarification in larger cohorts.

Donor characteristics may have contributed to the discrepant results between this study and the BDI score described by Op Den Dries et al. The BDI score was developed in a mixed donor population that included 23% of grafts from DCD donors [2]. Meanwhile, in our study, we have included only BDD grafts leading to a more homogenous donor population. When compared to BDD, DCD grafts experience additional warm ischaemia during organ retrieval leading to increased PVP injury and biliary strictures [2,32]. The selective inclusion of BDD grafts likely contributed to different rates of NAS (7% in this study vs. 19% in Op Den Dries et al.). Furthermore, in contrast to Op Den Dries et al., both NAS and AS were included in the primary analysis in this study due to the major role of ischaemic injury in the development of AS [5]. Histological injury was assessed in distal bile duct biopsies, adjacent to the biliary anastomosis, and these were presumed to reflect the entire biliary tree, as suggested by Op Den Dries et al. and Karimian et al. [2,33].

The limitations of this study include the small sample size and low incidence of biliary strictures, particularly NAS. The low incidence of biliary strictures was likely due to the exclusive analysis of BDD grafts, although the BDD sample size was similar to Op Den Dries et al.’s analysis [2]. Furthermore, as in the BDI score, the imaging of the biliary tree in this study was performed only when clinically indicated. Nevertheless, the results of this study suggest that the impact of mural stromal, vascular plexus and DPG injury in BDD grafts is less than previously reported. Furthermore, it should be acknowledged that WIT was significantly different between the biliary stricture group and the no-stricture group. Increased WIT may have exposed the biliary tree to increased injury, after the collection of the biopsy, and contributed to biliary strictures. However, it should be noted that the interval between hepatic artery and portal vein reperfusion, which is thought to have a greater role in the development of biliary strictures, was similar between groups [34]. It was also observed that donor age was higher in the biliary stricture group compared to the no-stricture group, similar to previous studies [6,7]. Advanced donor age is hypothesised to predispose grafts to increased ischaemia-reperfusion injury, resulting in stricture formation [7]. Despite an older donor population in this study, the incidence of biliary strictures in this study was lower than in Op Den Dries and Hansen et al. Conversely, although the biliary stricture group had younger recipients in this study, prior studies have not demonstrated any association between recipient age and biliary strictures [7,19]. Therefore, the role of recipient age in biliary strictures remains unclear and requires further clarification. Furthermore, all recipients received 24 months of follow-up to ensure biliary strictures were not missed, compared to the median follow-up of 17 months reported by Op Den Dries et al. Previous long-term studies have suggested that almost all biliary strictures would develop within 24 months, particularly those associated with injury during organ preservation [7,18].

In conclusion, the BDI score (mural stromal, PVP and DPG injury) was not correlated with the development of clinically relevant biliary strictures in this study. Therefore, our findings do not support the BDI score’s ability to predict biliary strictures in BDD grafts. Severe inflammation was the only histological feature (inversely) correlated with biliary strictures. Further studies are necessary to confirm and better understand this finding.

## Figures and Tables

**Table 1:** Definitions of Bile Duct Injury used by Op Den Dries et al. [2] and Hansen et al. [8].

	Grade 0	Grade 1	Grade 2	Grade 3
**Biliary Epithelial Injury**	No epithelial loss	≤50% loss	>50% loss	-
**Mural Stromal Injury**	No necrosis	≤25% necrotic	25–50% necrotic	>50% necrotic
**Peribiliary Vascular Plexus Injury**	No changes in vessels	≤50% of vessels with changes	>50% vessels with changes	>50% vessels with changes and arteriolonecrosis
**Thrombosis**	Absent	Present	-	-
**Intramural bleeding**	None	≤50% of duct wall	>50% of duct wall	
**Periluminal Peribiliary Gland Loss**	No cellular loss	≤50% loss of cells	>50% loss of cells	
**Deep Peribiliary Gland Loss**	No cellular loss	≤50% loss of cells	>50% loss of cells	
**Inflammation**	No inflammation	≥10 leucocytes/HPF	≥50 leucocytes/HPF	

Abbreviations: High-Powered Field.

**Table 2:** Demographic Characteristics.

		No-Stricture Group (n = 41)		Biliary Stricture Group (n = 16)		*p*-Value
		Median/Cases	IQR/%	Median/Cases	IQR/%	
**Donor Age**		54	43–61	61	55.5–68.3	0.018
Donor Risk Index *		1.581	1.413–1.1766	1.746	1.349–1.960	0.304
**Donor BMI**		26.9	23.7–29.1	23.9	22.7–26.9	0.051
**Donor Gender (M)**		19	46%	5	31%	0.378
**Cause of Donor Death**						0.122
**Trauma**		5	12%	2	13%	
**CVA**		20	49%	12	75%	
**Hypoxia**		16	39%	2	13%	
**Recipient Age**		60.5	55.6–64.01	52.3	47.7–60	0.026
**Recipient BMI**		29.1	24.3–34.1	27.7	25.7–37.9	0.534
MELD Score *		21	16–27	20	17–24	0.663
**Primary Transplant**		35	85%	13	81%	0.402
**Recipient Gender (M)**		27	66%	12	75%	0.752
**Recipient Liver Disease**						
**ETOH**		10	24%	7	44%	0.201
**HCV**		12	29%	5	31%	1.00
**NASH**		10	24%	3	19%	0.740
**Acute Liver Failure**		3	7%	1	6%	1.00
**PSC**		4	10%	1	6%	1.00
**HCC**		11	27%	5	31%	0.752
**CIT (min)**		325	261–389	209	273–386	0.894
**WIT (min)**		40	33–54	50	41–64	0.041
**Double bile duct flush**		17	41%	8	50%	0.570
**Duct-to-Duct Biliary Anastomosis**		32	78%	12	75%	1.00
**Interval between portal vein and hepatic artery revascularisation**		26	18–35	31	22–50	0.439
**Hepatic Artery Complication**		4	10%	5	31%	0.099
**Peak GGT levels within 7 days post-transplant**		247	149–345	388	283–527	0.083

* Abbreviations: Interquartile range (IQR), body mass index (BMI), cerebrovascular accident (CVA), model for end stage liver disease (MELD), alcoholic cirrhosis (ETOH), hepatocellular carcinoma (HCC), hepatitis C virus (HCV), hepatitis B virus (HBV), non-alcoholic steatohepatitis (NASH), Primary Sclerosing Cholangitis (PSC), cold ischaemic time (CIT), warm ischaemic time (WIT), Gamma-Glutamyl Transferase (GGT).

**Table 3:** Bile duct injury score stratified by development of biliary strictures.

		No-Stricture Group (n = 41)		Biliary Stricture Group(n = 16)		*p*-Value
	Grade	Cases	%	Cases	%	
**Biliary Epithelial Injury**	0	0	0%	0	0%	0.317
	1	9	22%	6	38%	
	2	32	78%	10	63%	
**Mural Stromal Injury**	0	11	27%	6	38%	0.700
	1	20	49%	5	31%	
	2	6	15%	3	19%	
	3	4	10%	2	13%	
**Peribiliary Vascular Plexus Injury**	0	0	0%	2	13%	0.158
	1	31	76%	11	69%	
	2	9	22%	3	19%	
	3	1	2%	0	0%	
**Thrombus**	0	38	93%	16	100%	0.552
	1	3	7%	0	0%	
**Intramural Bleeding**	0	34	83%	15	94%	0.420
	1	7	17%	1	6%	
	2		0%		0%	
Peribiliary Glands *	0	6	15%	5	33%	0.261
	1	24	60%	6	40%	
	2	10	25%	4	27%	
Deep Peribiliary Gland *	0	10	26%	5	36%	0.831
	1	23	59%	7	50%	
	2	6	15%	2	14%	
**Inflammation**	0	12	29%	8	50%	0.003
	1	12	29%	8	50%	
	2	17	41%	0	0%	

* periluminal peribiliary glands and deep peribiliary glands were not seen in specimens 2 and 4, respectively.

**Table 4:** Bile duct injury scores stratified by development of Non-Anastomotic Strictures.

		No-NAS Group (n = 53)		Non-Anastomotic Stricture Group (n = 4)		*p*-Value
	Grade	Cases	%	Cases	%	
**Biliary Epithelial Injury**	0	0	0%	0	0%	1.00
	1	14	26%	1	17%	
	2	39	74%	3	50%	
**Mural Stromal Injury**	0	16	30%	1	17%	0.682
	1	23	43%	2	33%	
	2	9	17%	0	0%	
	3	5	9%	1	17%	
**Peribiliary Vascular Plexus Injury**	0	1	2%	1	17%	0.166
	1	40	75%	2	33%	
	2	11	21%	1	17%	
	3	1	2%	0	0%	
**Thrombus**	0	50	94%	4	67%	1.00
	1	3	6%	0	0%	
**Intramural Bleeding**	0	46	87%	3	50%	0.464
	1	7	13%	1	17%	
	2		0%			
Peribiliary Glands *	0	10	20%	1	17%	0.390
	1	29	57%	1	17%	
	2	12	24%	2	33%	
Deep Peribiliary Gland *	0	14	29%	1	17%	0.792
	1	28	57%	2	33%	
	2	7	14%	1	17%	
**Inflammation**	0	17	32%	3	50%	0.313
	1	19	36%	1	17%	
	2	17	32%	0	0%	

* periluminal peribiliary glands and deep peribiliary glands were not seen in specimens 2 and 4, respectively.

## Data Availability

The data presented in this study are available on request from the corresponding author.
